# A Mathematical Model Coupling Tumor Growth and Angiogenesis

**DOI:** 10.1371/journal.pone.0149422

**Published:** 2016-02-18

**Authors:** Jiangping Xu, Guillermo Vilanova, Hector Gomez

**Affiliations:** Applied Mathematics, University of A Coruña, A Coruña, Spain; Rensselaer Polytechnic Institute, UNITED STATES

## Abstract

We present a mathematical model for vascular tumor growth. We use phase fields to model cellular growth and reaction-diffusion equations for the dynamics of angiogenic factors and nutrients. The model naturally predicts the shift from avascular to vascular growth at realistic scales. Our computations indicate that the negative regulation of the Delta-like ligand 4 signaling pathway slows down tumor growth by producing a larger density of non-functional capillaries. Our results show good quantitative agreement with experiments.

## Introduction

### Aims and Scope

**Fig 1:**
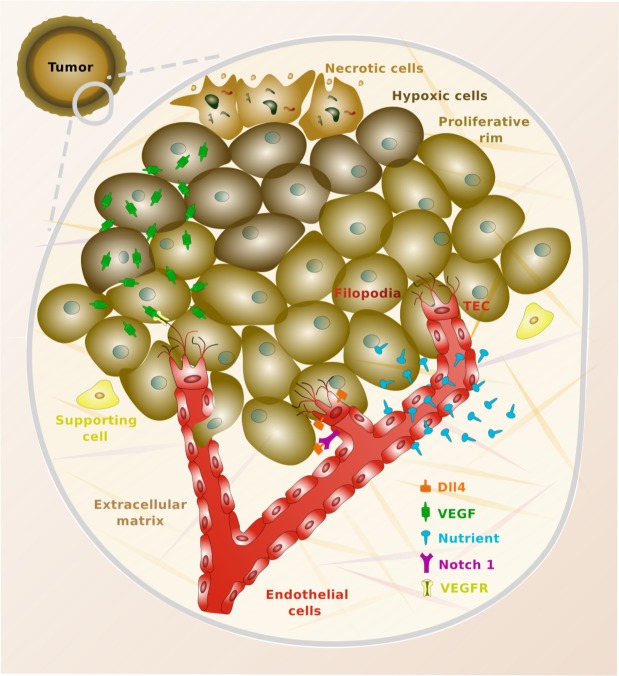
Vascular tumor growth. The top left part represents a relatively small tumor which is undergoing the vascular switch. The tumor is radially divided into three parts, which represent the proliferative rim (outer ring), the hypoxic zone (mid part), and the necrotic core (inner area). A small area of the tumor is surrounded by a gray solid line whose interior is zoomed in. In the zoomed-in area, we can observe tumoral cells colored with different brown tones, according to their condition of necrotic, hypoxic, or proliferative cells. The red area shows the capillaries, which are lined by endothelial cells as shown in the plot. The new sprouts are led by tip endothelial cells (TECs), which are endowed with filopodia to better explore their surroundings [10, 15]. The picture also shows how the capillaries release nutrients that diffuse throughout the tissue. Similarly, hypoxic cells release vascular endothelial growth factor (VEGF), which eventually binds to surface receptors located in the membrane of endothelial cells (VEGFR). Finally, the figure shows how TECs overexpress the protein Delta-like ligand 4 (Dll4). This protein binds to the Notch receptors of nearby endothelial cells, preventing them from also becoming TECs [12, 13].

Tumor growth is governed by complex biological mechanisms that occur at different scales. Arguably, the most representative scale partition is that defined by the molecular, cellular, and tissue scales. Examples of relevant phenomena taking place at molecular scale include regulation of signaling pathways at the cell membrane, nutrient uptake, or the degradation of DNA that leads to abnormal proliferation of tumor cells. Several cell activities, such as division, necrosis, and apoptosis occur at cellular level. The boundary between cellular and tissue scales is blurry, but we consider the tissue scale to be operative when the tumor is able to trigger angiogenesis. Angiogenesis is the formation of new capillaries from pre-existing vasculature and is a crucial step for a tumor to become a health problem. In fact, it has been shown that tumors unable to induce angiogenesis cannot grow larger than 1 mm in radius approximately [1]. In the avascular stage of tumor growth, that is, prior to the recruitment of new vasculature, the tumor feeds on nutrients that diffuse to its surface. Tumors consume much more nutrients than healthy cells and the nourishment cannot penetrate deep into the lesion. This produces a heterogeneous distribution of nutrients inside the tumor, primarily controlled by the distance to the tumor surface. On the basis of this nutrient distribution, the tumor may be divided into three regions, namely, the necrotic core, the hypoxic zone, and the proliferative rim (see Fig 1). In the proliferative rim, cells have enough nutrient to divide rapidly, while in the necrotic core the nutrient levels are so low that cells die from starvation, generating necrotic debris that remains inside the tumor due to the scarcity of lymphatic and blood capillaries. In the hypoxic zone, however, tumor cells become temporarily quiescent and eventually apoptotic if no additional nutrient becomes available. To circumvent this situation, hypoxic tumor cells secrete growth factors that precipitate angiogenesis. Well-known examples of these factors are vascular endothelial growth factor (VEGF, [2]), basic fibroblast growth factor (bFGF, [3]), angiopoietin 2 (Ang-2, [4]), thrombospondin-1 (TSP-1, [5]), or chemokines [6]. While some of them are pro-angiogenic and promote proliferation and migration of endothelial cells, others are anti-angiogenic and favor vessel stability and endothelial cell dormancy. Under physiological conditions, their overall effect maintains endothelial cell homeostasis and prevent angiogenesis. The tumor angiogenic factors released by hypoxic tumor cells, however, disturb this equilibrium between pro- and anti-angiogenic factors and trigger the angiogenesis cascade [7]. Although the angiogenesis process is exceedingly complex (see [8, 9] for reviews), at this stage, we will just say that the new capillaries grow towards the tumor, mainly following the signals of the growth factors. Capillaries are lined by endothelial cells and the growth of new sprouts requires these cells to migrate and proliferate. In fact, when tumor-produced growth factors reach endothelial cells they differentiate into two phenotypes, namely, stalk endothelial cells (SECs) and tip endothelial cells (TECs), which are associated to the proliferative and migratory cells, respectively [10, 11]. More specifically, during angiogenesis, VEGF and Notch signaling pathways are involved in the differentiation of TECs and SECs in the vascular endothelium. Under the stimulation of VEGF, the expression of Delta-like ligand 4 (Dll4) is up-regulated in TECs. Dll4 binds to Notch receptors of nearby endothelial cells which, in turn, reduces their VEGF-receptor expression, consequently suppressing their TEC phenotype [12, 13]. Experimental evidence shows that TECs lead to sprouting vessels and migrate along the gradient of tumor-produced growth factors while adjacent SECs generate the trunk of the new vessels and maintain connectivity with the parental vessels [14]. Furthermore, TECs develop slender cytoplasmatic protrusions called filopodia that survey the cell microenvironment and facilitate the migration [10, 15]. After new capillaries are formed, the vascular network gradually remodels and evolves to achieve successful functionality. When the tumor becomes well-vascularized, the new blood vessels provide cancerous cells with sufficient nutrients and a means to escape and colonize other tissues, which is potentially very harmful. Some tumor types are more vascularized than others. For example, prostatic carcinomas are usually highly vascularized, while lung carcinomas are not [16]. However, even well-vascularized tumors are riddled with regions subjected to acute and chronic hypoxia [17]. It may also be surprising that if the tumor angiogenesis process is altered by permitting the growth of more sprouts than would naturally be formed, the tumor becomes more hypoxic and grows slower [18]. Our model tries to address these queries and establish a predictive tool that can pose new questions to be probed experimentally.

From a technical point of view, one of the most difficult obstacles to develop a model for coupled tumor growth and angiogenesis is the need to bridge, at least to some extent, the cellular and tissue scales. This may be one of the reasons why most existing models deal with either vascular or avascular growth, but do not account for both phenomena in the same theory. A related difficulty stems from the fact that tumor growth has been primarily modeled using continuum models, while discrete methods prevail in angiogenesis modeling. Here, we present a model in which capillaries are resolved to full scale and interact with tumors in a fully-coupled manner.

### A Brief Bibliographic Survey

Early models of avascular growth tried to reproduce the features observed in multicellular tumor spheroids [19], in particular the differentiation between the proliferative rim, the hypoxic zone, and the necrotic core. Chaplain and Britton presented a predictive model to examine the steady-state profile of the growth inhibitory factor in spheroids [20], and Byrne and Chaplain studied the stability of steady, radially-symmetric solutions with respect to perturbations [21, 22] by assuming the three distinct layers. However, detailed experimental investigations showed that the transitions between the three layers could be gradual, rather than sharp [23]. This led to mathematical models in which the layers were defined by continuum densities of proliferative, quiescent, and dead cells [24, 25]. Some other models of avascular growth focus on reproducing different features, such as, for example growth saturation or cell movement [26–29].

From a methodological point of view, the mixture theory has been a useful framework within the continuum modeling of avascular growth [30–32]. More recently, a new approach based on the multiphase porous media mechanics theory, has also been proposed [33–35]. Another framework which has produced very relevant results is that provided by the phase-field method [36–41], which we also employ here. The literature is also rich on discrete models belonging to the class of cellular automata or agent-based methods that have tried to model avascular growth by considering cell-cell interactions, cell-matrix interactions, and the effect of the microenvironment [42–44].

Vascular growth modeling has attracted significant interest in the last few years. Prime examples include the work of Frieboes *et al* [45, 46], which couples phase fields to describe tumor growth with discrete random walks to model angiogenesis. We also mention the ten-species model of Lima *et al* [47], which was recently proposed. Multiscale techniques have also proven important on the development of vascular growth theories, as shown in [48]. Significant examples within the cellular automata framework were given in [49–51]. Other important work includes [52–58].

## Methods

### Mathematical Model

In this section, we propose a phenomenological model for the coupled dynamics of tumor growth and angiogenesis. Our model is chiefly continuous, although it also involves discrete agents representing TECs. The discrete agents are seamlessly integrated as part of one of the continuous fields using the concept of template functions [59]. This yields a virtually continuous model, whose solution may be numerically approximated using a partial-differential equation (PDE) solver with minor changes only. For the purely-continuous part of our model, we will use classical reaction-diffusion equations to describe the dynamics of chemical substances and phase-field equations to model tumor and capillary growth. The phase-field theory is a mathematical formalism to derive models for problems with moving interfaces [60–62]. Phase fields may be thought of as smooth functions, which act as markers of the location of a feature of interest, where the word feature should be understood in a broad sense. In classical applications of phase fields, e.g., in mechanics, the phase field may define, for instance, the location of a particular component in multicomponent flows or the spatial distribution of crystallites (grains) in a polycrystalline material. Here, we use phase fields as markers of the location of different cell types. In particular, we use two phase fields, one to mark the location of tumor cells (*ϕ* ∈ [0, 1]) and another one to define the position of endothelial cells (*c* ∈ [−1, 1]). Also within the purely continuous description, we use two additional functions, namely, *σ* and *f*. The function *σ* ∈ [0, 1] represents the concentration of a generic substance, which is assumed to drive tumor growth. This quantity may play the role of a vital nutrient (e.g., oxygen or glucose), a growth factor, or another chemical controlling tumor proliferation. For simplicity, in what follows, we will refer to *σ* as nutrient concentration. The function *f* ∈ [0, 1] defines the concentration of a generic substance which represents the balance between the pro- and anti-angiogenic factors. This conceptualization simplifies the biochemical interactions that take place in tumor angiogenesis, in which a number of substances released by hypoxic tumor cells reach the surface receptors of endothelial cells. Some of these chemicals favor angiogenesis (e.g., VEGF or bFGF), while others preclude the generation of new sprouts in one way or another (e.g., TSP-1 or Ang-2). The net result of these intricate molecular mechanisms (see [7] for details) is that hypoxic cells are able to pull endothelial cells out of their tightly-controlled homeostatic condition by unbalancing the angiogenic factor equilibrium towards angiogenesis [9]. Thus, the unknown *f* represents the net pro-angiogenic contribution of all these substances. In what follows, we will simply refer to *f* as tumor angiogenic factor (TAF). Our model also makes use of discrete agents which represent TECs. Due to their migratory phenotype, TECs lead the way of new sprouts following cues of different types. The template functions that we use are simple approximations to a phase field that represents the location of an individual cell [59, 63]. The discrete elements are mesh-free in the sense that they are independent from the spatial discretization. Due to the migratory nature of TECs, the discrete agents are advected using a suitable velocity. Essentially, the velocity depends on chemotactic cues and certain conditions on the microenvironment of the cell, which replicates the fact that TECs explore their surroundings using filopodia. All the details about the governing equations are given in what follows.

#### Tumor (*ϕ*)

The tumor location is described by a PDE governing the evolution of the phase field *ϕ*. As we will explain later, the equation governing *ϕ* naturally produces smooth but thin transitions between two constant states which identify cancerous and host tissue. As a consequence of the use of phase fields in the model, the tumor dynamics occurs mainly at the interface between malignant and host cells. We believe this hypothesis is plausible, since in non-invasive solid cancer, tumoral cells cluster forming highly-packed masses with a well-defined interface. Also, due to the scarcity of both lymphatic and blood vessels inside the tumor, it seems reasonable to assume that the dynamics occurs mainly at the interface. The usual starting point to derive a phase-field equation is a free-energy functional. In this study, we define the tumor free energy as

$$\begin{array}{c} \Psi_{\phi }\left(\phi ,\nabla \phi ,\sigma \right)=\Psi_{\phi }^{\text{s}}\left(\nabla \phi \right)+\Psi_{\phi }^{\text{ch}}\left(\phi ,\sigma \right)\text{,} \end{array}$$ (1)

where $\Psi_{\phi }^{\text{s}}$ is the tumor surface free energy and $\Psi_{\phi }^{\text{ch}}$ is the tumor chemical free energy. The phase-field equation is defined in such a way that, if the nutrient concentration is kept fixed, the free energy Ψ_*ϕ*_ decreases with time along solutions of the equation. This procedure is often referred to as gradient dynamics [64]. In Eq (1), the tumor surface free energy is defined as

$$\begin{array}{c} \Psi_{\phi }^{\text{s}}\left(\nabla \phi \right)=\frac{1}{2}\lambda_{\phi }^{2}{\left|\nabla \phi \right|}^{2}, \end{array}$$ (2)

where *λ*_*ϕ*_ is a positive constant proportional to the interface width and |⋅| denotes the magnitude of a vector. The tumor chemical free energy is given by

$$\begin{array}{c} \Psi_{\phi }^{\text{ch}}\left(\phi ,\sigma \right)=g\left(\phi \right)+m\left(\sigma \right)h\left(\phi \right), \end{array}$$ (3)

where

$$\begin{array}{c} g\left(\phi \right)=\phi^{2}{\left(1-\phi \right)}^{2};\;h\left(\phi \right)=\phi^{2}\left(3-2\phi \right);\;m\left(\sigma \right)=\frac{-2}{3.01\pi }\arctan\left(15\left(\sigma -\sigma^{h-v}\right)\right)\text{.} \end{array}$$ (4)

**Fig 2:**
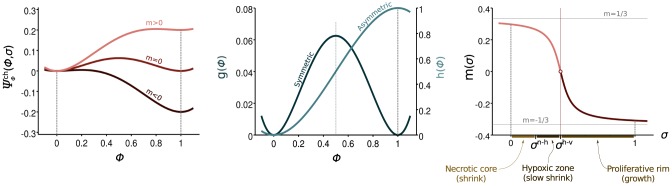
Chemical free energy of the tumor. The tumor chemical free energy, $\Psi_{\phi }^{\text{ch}}$, is defined as a double-well potential formed by the sum of a symmetric contribution (*g*) and a non-symmetric function (*h*). The latter is multiplied by a tilting function *m*, that depends on the nutrient concentration. On the right hand side of the plot, *σ*^*n*−*h*^ and *σ*^*h*−*v*^ represent values of the nutrient concentration that define, respectively, the necrotic-hypoxic and hypoxic-viable thresholds.

Here, *σ*^*h*−*v*^ represents the value of the nutrient concentration that defines the threshold between hypoxic and viable tumor cells. The left panel of Fig 2 shows a plot of $\Psi_{\phi }^{\text{ch}}$, whereas the middle and right panels show plots of *g*(*ϕ*), *h*(*ϕ*), and *m*(*σ*). Note that *g*(*ϕ*) is a symmetric function with two local minima. Functions with this structure are typically referred to as double-well potentials. The term *m*(*σ*)*h*(*ϕ*) introduces a non-symmetric perturbation in $\Psi_{\phi }^{\text{ch}}$, whose magnitude depends on the value of the nutrient concentration, as shown in Fig 2. The function *m*(*σ*) is usually called tilting function and must verify the condition |*m*(*σ*)| < 1/3. For the particular form of *m* that we use, this implies that the denominator in front of the arctan must be greater than 3*π*. Although other choices are possible, for simplicity we just take a value of 3.01*π*. The specific form of *m* has little impact on the results (see [62] for details). The fulfillment of the condition |*m*(*σ*)| < 1/3 guarantees that the potential $\Psi_{\phi }^{\text{ch}}$ achieves local minima at *ϕ* = 0 and *ϕ* = 1 for all *σ* > 0. This suggests that the solution will be driven to these constant states, which represent, respectively, the host and cancerous tissue. Note, however, that the energy levels achieved at the local minima are different from each other when *σ* ≠ *σ*^*h*−*v*^. When *σ* < *σ*^*h*−*v*^ (i.e., *m* > 0) the energy level is lower at *ϕ* = 0, and as a consequence, the host tissue is energetically preferred. When *σ* > *σ*^*h*−*v*^ (i.e., *m* < 0), the opposite situation occurs and the phase-field equation will favor tumor growth. Using the tumor free energy and the concept of gradient dynamics, we derive the following non-conserved phase-field equation for the tumor evolution

$$\begin{array}{c} \frac{\partial \phi }{\partial t}=M_{\phi }\left(\lambda_{\phi }^{2}\Delta \phi -\mu_{\phi }\left(\phi ,\sigma \right)\right)\text{,} \end{array}$$ (5)

where $\mu_{\phi }=\partial \Psi_{\phi }^{\text{ch}}/\partial \phi$, and *M*_*ϕ*_ is a positive constant that represents the tumor mobility. Eq (5) controls the tumor dynamics, which is strongly dependent on the nutrient distribution. The nutrient, in turn, is released by capillaries whose location depends, among other things, on the TAF distribution. This makes the problem fully coupled.

#### Nutrient (*σ*)

We assume that the nutrient is supplied by capillaries, diffuses throughout the tissue, and is consumed by tumor and host cells at a different rate. This is modeled by the reaction-diffusion equation

$$\begin{array}{c} \frac{\partial \sigma }{\partial t}=\nabla \cdot \left(D_{\sigma }\nabla \sigma \right)+V_{p}^{c}\left(1-\sigma \right)cH\left(c\right)S-V_{u}^{T}\sigma \phi -V_{u}^{H}\sigma H\left(1-\phi \right)\text{,} \end{array}$$ (6)

where *D*_*σ*_ is the nutrient diffusion constant, $V_{p}^{c}$ is the nutrient production rate at the capillaries, H(·) is a smoothed-out Heaviside step function, while $V_{u}^{T}$ and $V_{u}^{H}$ are the uptake rates of nutrient by cancerous and host tissue, respectively. A common assumption that we also adopt here is that $V_{u}^{T}>>V_{u}^{H}$ (see Table 1). The consumption term $V_{u}^{H}\sigma H\left(1-\phi \right)$ implicitly assumes that the host tissue and the capillaries have the same uptake rate of nutrient. Finally, the dimensionless quantity S is a crude measure of the structure and density of the capillary network. Assuming that at a given time, the vasculature in the tissue is composed by *n* capillaries of lengths ${\left\{l_{i}\right\}}_{i=1}^{n}$, the value of S is given by

$$\begin{array}{c} S=\frac{l_{\text{max}}}{\sum_{i=1}^{n}l_{i}},\;\text{where}\;l_{\text{max}}=\max_{1\leq i\leq n}\left(l_{i}\right)\text{.} \end{array}$$ (7)

The expression of S given by Eq (7) is often used in experimental research on angiogenesis to characterize a capillary network [65]. Data indicate that, as the network becomes more intricate, the value of S decreases. In our model, this implies that the nutrient production is effectively reduced when the vasculature becomes denser [see Eq (6)], a fact supported by abundant experimental evidence [18]. Although this observation is not completely understood, the reduced nutrient production of intricate networks is often attributed to the leakiness and tortuosity of dense, tumor-induced vascular networks.

**Table 1:** *In silico* values of the parameters used in the proposed model.

Parameter	Symbol	*In silico* value
Diffusion coefficient of the tumor	*M*_*ϕ*_	0.3
Interface width of tumor	*λ*_*ϕ*_	$2\sqrt{2}$
Diffusion coefficient of the nutrient	*D*_*σ*_	30
Production rate of nutrient	$V_{p}^{c}$	1
Uptake rate of nutrient by tumor	$V_{u}^{T}$	6 × 10^−3^
Uptake rate of nutrient by host tissue	$V_{u}^{H}$	6 × 10^−4^
Necrotic/hypoxic-cell threshold	*σ*^*n*-*h*^	0.2
Hypoxic/viable-cell threshold	*σ*^*h*-*v*^	0.4
TAF condition for highest proliferation	*f*_*p*_	0.3
Mobility of capillaries	*M*_*c*_	1
Interface width of capillaries	*λ*_*c*_	1
Proliferation rate of endothelial cells	*B*_*p*_	1.401
TEC radius	*R*	4
Condition 1 for TEC (de)activation	*c*_*act*_	0.9
Condition 2 for TEC (de)activation	*f*_*act*_	0.001
Chemotatic constant	*χ*	7.28
Dll4 effective distance	*δ*_4_	80
Diffusion coefficient of TAF	*D*_*f*_	100
Uptake rate of TAF by capillaries	*B*_*u*_	6.25

Parameters related to angiogenesis are obtained from [59].

#### Capillaries (*c*)

Capillaries are defined by a phase-field *c* which maps the concentration of endothelial cells to [−1, 1] and implicitly defines their location. The phase-field equation that governs *c* naturally develops areas in which *c* = 1 (tissue occupied by capillaries) and *c* = −1 (capillary-free tissue). Following prior work [59, 66], we model the evolution of the phase-field *c* using the equation

$$\begin{array}{c} \frac{\partial c}{\partial t}=\nabla \cdot \left(M_{c}\nabla \left(\mu_{c}\left(c\right)-\lambda_{c}^{2}\Delta c\right)\right)+B_{p}\left(f\right)cH\left(c\right)\text{,} \end{array}$$ (8)

where *M*_*c*_ is the mobility of the capillaries, *λ*_*c*_ is a positive constant related to the width of the capillary wall, $B_{p}\left(f\right)$ is the proliferation rate of endothelial cells as a function of the TAF concentration *f*, and *μ*_*c*_(*c*) = *c*^3^−*c* is the derivative of the double-well potential

$$\begin{array}{c} \Psi_{c}^{\text{ch}}\left(c\right)=\frac{c^{4}}{4}-\frac{c^{2}}{2}. \end{array}$$ (9)

Note that $\Psi_{c}^{\text{ch}}$ is a symmetric double-well potential with two local minima at *c* = −1 and *c* = 1. Therefore, Eq (8) resembles a Cahn-Hilliard-type equation [67] with a reaction term that depends on the TAF concentration. In particular, inside the capillaries, where *c* ≈ 1, the reaction term reduces to

$$\begin{array}{c} B_{p}\left(f\right)=\{\begin{array}{cc} B_{p}f & \text{if}\;f<f_{p}\\ B_{p}f_{p} & \text{if}\;f\geq f_{p} \end{array}\text{,} \end{array}$$ (10)

where *f*_*p*_ is a constant representing a threshold value at which proliferation saturates to its maximum rate defined by *B*_*p*_ *f*_*p*_. Eq (8) may be thought of as the model that controls the dynamics of stalk endothelial cells, which are those with a proliferative phenotype. As anticipated before, TECs, which have a migratory phenotype, are modeled separately using discrete agents that we eventually integrate in the field *c*.

#### Tip endothelial cells (TECs)

The tumor angiogenic factor can drive a phenotypic change in endothelial cells when it binds to their surface receptors. In fact, some privileged cells (TECs) acquire a migratory phenotype and lead the growth of new capillaries. It is known that in order to create functional networks with a moderate amount of sprouts, the number of tip endothelial cells in a region is negatively regulated by a factor called Delta-like ligand 4 (Dll4) [68]. This factor is overexpressed by TECs and binds to the Notch receptors of nearby endothelial cells, preventing them from becoming tip endothelial cells (see Fig 1). Hence, only a selection of endothelial cells generate new sprouts. The remaining endothelial cells affected by tumor angiogenic factor become proliferative, and their dynamics is controlled by Eq (8). For supporting experimental evidence, the reader is referred to [10, 11, 14].

TECs are characterized by protrusions called filopodia through which they probe the conditions of their microenvironment (see, e.g., [69]). The expression of tumor angiogenic factor receptors is higher on filopodia than in regular cell membrane. Consequently, filopodia enhance the tip endothelial cell chemical sensitivity towards TAF and facilitate their migration following chemotactic cues. In addition, filopodia permit tip endothelial cells to detect nearby capillaries [15]. There is experimental evidence showing that when a nearby capillary is detected, tip endothelial cells alter their migration towards them to form loops through anastomoses that eventually enhance blood flow and tissue oxygenation. For these reasons, tip endothelial cells are modeled here as circular (with radius *R*), mesh-free, discrete agents that can get activated and deactivated, spread filopodia, move following chemotactic gradients of tumor angiogenic factor, detect nearby capillaries, and anastomose with them. In particular, a new tip endothelial cell is activated (deactivated) if the following conditions are met (not met) at a point:

1. *c* ≥ *c*_*act*_, which guarantees that the point is inside a capillary;
2. *f* ≥ *f*_*act*_, which establishes the minimum TAF concentration to trigger the activation; and
3. the distance to any other tip endothelial cell is larger than *δ*_4_, which assures sparser, more functional networks.

The last condition represents a simple model of the Delta/Notch signaling pathway. By preventing TECs to be formed in the surroundings of another active tip cell, we encode the essential mechanism whereby Dll4 controls the density of the vascular network. In what follows, we call *δ*_4_ effective Dll4 distance. Note that the process is deterministic, except for a rather insignificant detail. Whenever exist several points satisfying conditions 1, 2, and 3 listed above we choose one randomly.

When the above conditions are met, active TECs migrate with a velocity given by the expression

$$\begin{array}{c} v=\chi \frac{\nabla f}{\left|\nabla f\right|}J\left(\phi \right),\;\text{where}\;J\left(\phi \right)=0.45\left[\text{tanh}\left(50\left(0.5-\phi \right)\right)+1\right]+0.1\text{.} \end{array}$$ (11)

Here, *χ* is the chemotactic constant. The velocity **v** follows gradients of TAF and J(ϕ) is a function that takes values in the interval (0, 1) and incorporates the observation that capillaries can penetrate the tumor, but they do so at a velocity significantly smaller than that of TECs migrating in the surrounding tissue. The function J(ϕ) may be thought of as a translated, scaled, and smoothed-out Heaviside function. For large *ϕ* (inside the tumor), J(ϕ) takes approximately the value 0.1, while for small *ϕ* (outside the tumor) it is approximately equal to 1. For intermediate values, J(ϕ) provides a quick, but smooth transition between 0.1 and 1.

In addition, once TECs have migrated more than four times their radius away from their parent vessel, the value of *c* is checked at a set of points that mimic filopodia. If any of these values is positive, then a capillary is detected and the TEC migrates in that direction until it anastomoses and gets deactivated [70].

#### Tumor angiogenic factor (*f*)

In our model, TAF is released by hypoxic malignant cells, diffuses throughout the tissue, and is consumed by endothelial cells, as expressed by the reaction-diffusion equation

$$\begin{array}{c} \frac{\partial f}{\partial t}=\nabla \cdot \left(D_{f}\nabla f\right)+\phi \left(1-f\right)G\left(\sigma \right)-B_{u}fcH\left(c\right)\text{.} \end{array}$$ (12)

**Fig 3:**
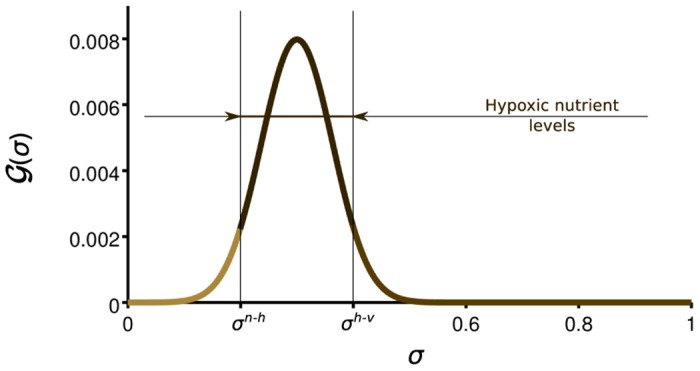
Secretion rate of tumor angiogenic factor (G(σ)). Tumor angiogenic factor is released by hypoxic tumor cells, that is, those whose nutrient availability is not enough for proliferation, but higher than the apoptotic threshold (*σ*^*n*-*h*^ > *σ* > *σ*^*h*-*v*^).

Here *D*_*f*_ is the diffusion constant, *B*_*u*_ is the uptake rate of TAF by capillaries, and G is the secretion rate of tumor angiogenic factor released by tumor cells. Since TAF is chiefly released by hypoxic tumor cells, we define G as a hill function of *σ*. More specifically,

$$\begin{array}{c} G\left(\sigma \right)=\frac{0.02}{\sqrt{2\pi }}\exp\left(-125{\left(\sigma -\frac{\sigma^{n\text{-}h}+\sigma^{h\text{-}v}}{2}\right)}^{2}\right)\text{,} \end{array}$$ (13)

where *σ*^*n*-*h*^ is the value of the nutrient concentration that defines the threshold between necrotic and hypoxic tumor cells. Fig 3 shows a plot of G and illustrates how TAF is mainly released in the hypoxic region of the tumor, that is, where the nutrient concentration is between *σ*^*n*-*h*^ and *σ*^*h*-*v*^.

### Computational Method

The numerical solution of the equations that govern our model poses significant computational challenges due to strong non-linearities, stiffness in space and time, and the presence of fourth-order derivatives. Although the equations can be solved on square geometries using classical finite-difference methods, we employ highly-efficient algorithms that will eventually permit us to perform three-dimensional computations on larger-scale, realistic tissue geometries. Our computational technology is based on isogeometric analysis [71], a recent generalization of the finite element method that excels by its two- and three-dimensional geometric flexibility, robustness, and higher-order accuracy and continuity [72, 73]. Isogeometric analysis has proven a very efficient technology to discretize differential equations involving higher-order operators and giving rise to thin layers in the solution [74–76], which are challenges similar to those posed by our current model for vascular tumor growth. In particular, we benefit from isogeometric analysis using $C^{1}$-continuous basis functions, which permit a straightforward discretization of fourth-order differential operators without introducing additional degrees of freedom [77, 78]. To perform the time discretization, we use the generalized-*α* algorithm, which is a second-order accurate and *A*-stable method with optimal dissipation properties [79]. To speed up the computations, we use an adaptive time-step selection algorithm based on the ideas presented in [80, 81].

Finally, the discrete agents representing TECs are seamlessly integrated with the phase field that defines capillaries using the concept of template functions. The phase-field is just a marker of the location of capillaries and the agents represent individual cells which are assumed to have circular shape. Therefore, we define simple approximations to a phase field representing a single circular cell and use that as a template which moves following TECs. Our template functions are smoothed-out radial Heaviside functions which take approximately the value +1 inside the circular cell and −1 elsewhere. Using these ideas, our model can be solved numerically using a standard PDE solver with minor changes only.

## Results

Here, we present numerical results which illustrate two important features of our model. First, we show that our theory naturally predicts the shift from avascular to vascular growth by triggering angiogenesis. This example also shows that if angiogenesis is blocked, the tumor reaches a maximum size, and then gradually regresses, as shown in experiments [1]. Second, our model reproduces a recently-observed phenomenon in vascularized tumors in which cancer growth is hindered by down-regulating the Delta/Notch pathway. This hampers the control that Dll4 exerts on the creation of new TECs, leading to denser and more inefficient vascular networks, which in turn give rise to smaller tumors.

### Simulation Setup

**Fig 4:**
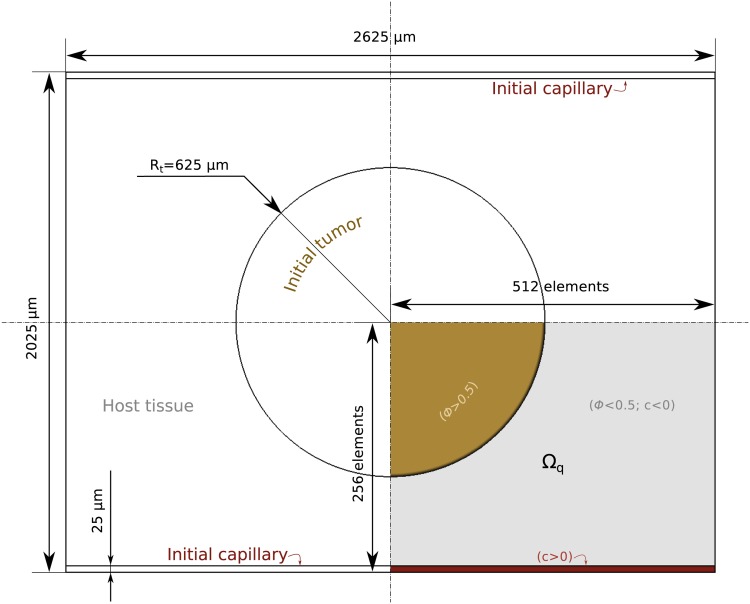
Problem setup. The domain of the problem represents a rectangular tissue of size 2625 µm × 2025 µm with a circular initial tumor (brown) and two capillaries (red). Using the symmetries of this setup, the simulations may be performed on a quarter of the tissue, the domain Ω_*q*_.

To perform the computations, we non-dimensionalize the equations using a length scale *L* = 1.25 µm and a time scale *T* = 1562.5 s. These scales may be used to find the values of the physical parameters from the *in silico* quantities reported in Table 1, which are the values used in the computations. We perform our computations on a rectangular tissue of 2625 µm × 2025 µm. We place an initial circular tumor at the center of the domain and two horizontal capillaries in the bottom and top boundaries, as shown in Fig 4. Boundary conditions are also taken to maintain symmetry, so that we can perform the computations on a quarter of the domain Ω_*q*_ (bottom right quadrant). The mesh is composed of 512 × 256 quadratic elements. The radius of the initial tumor is *R*_*t*_ = 625 µm and the capillaries are supposed to be initially straight with a constant width of 25 µm. Therefore, the value of *c* is set to −1 everywhere in Ω_*q*_, except in the stripe region at the bottom, where is set to 1. The size of the initial tumor corresponds to that of a typical human carcinoma which can no longer feed on the nutrients that diffuse to its surface and has to trigger angiogenesis to grow further. The initial source of nutrient is the capillary at the bottom, where *σ* takes the value 1. We assume that, initially, the tumor has a circular necrotic core concentric to the tumor and with a radius *R*_*n*_ = 0.45*R*_*t*_, where *σ* = 0. Everywhere else in the tissue, *σ* = 0.45.

### Avascular Versus Vascular Tumor Growth

**Fig 5:**
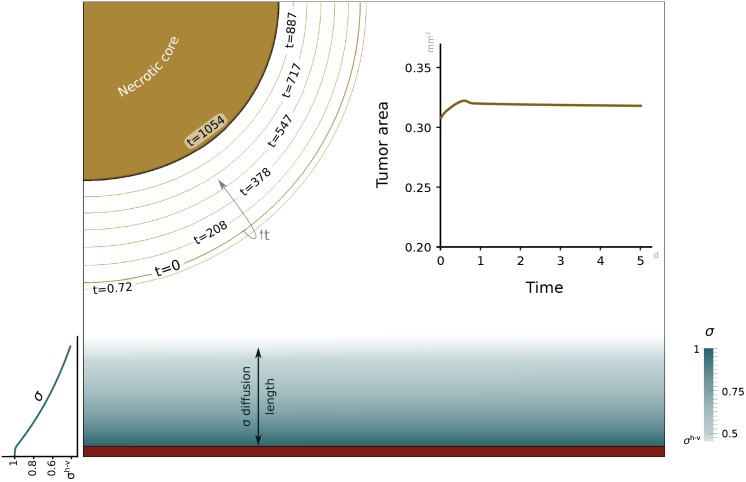
Simulation of avascular tumor growth. The amount of nutrient (blue scale) released from the capillary at the bottom edge is not enough for tumor cells to proliferate. Thus, the tumor remains at a constant size within usual experimental time scales (tumor area graph) and shrinks at very long time scales (brown contour lines). By day 1054 (solid brown), the tumor radius has been reduced to approximately 67% of its initial value.

In this example, we initially simulate the blockade of angiogenesis by freezing Eqs (8) and (12), and not allowing the activation of TECs. As a consequence, the vascular network reduces to the initial capillary, which is the only source of nutrient. It may be easily seen that, in this case, S=1 throughout the simulation. Fig 5 shows the solution at time *t* = 1054 days. The top left part of the figure, which corresponds to the tumor, is accompanied by contour lines of the tumor interface at different times to observe the evolution of the lesion. On the top right, the inset shows the time evolution of the tumor area. It may be observed that the tumor grows slightly at the beginning and then regresses slowly due to its inability to trigger angiogenesis. The initial growth is small (∼10%) and occurs at short time scales. It is a consequence of the initial nutrient distribution, which is just a crude approximation to the actual one. The eventual regression occurs at very long time scales, but at usual experimental time scales (see the top-right inset in Fig 5) the tumor volume remains constant. In the top-right inset, we have used the same time span that will be employed in the vascular-growth simulation, for comparison purposes. However, the contour lines on the top-left figure show that the tumor volume remains fairly constant for intermediate time scales (see, for example, the contour line for *t* = 208 days). Thus, our results are consistent with the experimental observation indicating that the tumor becomes dormant when is unable to trigger angiogenesis. The bottom part of Fig 5 shows the nutrient distribution at time *t* = 1054 days. At this point, the nutrient has achieved a quasi-steady configuration, which corresponds to the expected situation. In particular, the nutrient is maximum at the source and then decreases gradually with the distance to the capillary. It may be observed that the threshold value *σ*^*h*−*v*^ is achieved at a distance to the capillary of approximately 200 µm, which is consistent with abundant experimental evidence. The nutrient concentration further away from the capillary is lower than *σ*^*h*−*v*^, and in particular, is very low inside the tumor as one would expect.

**Fig 6:**
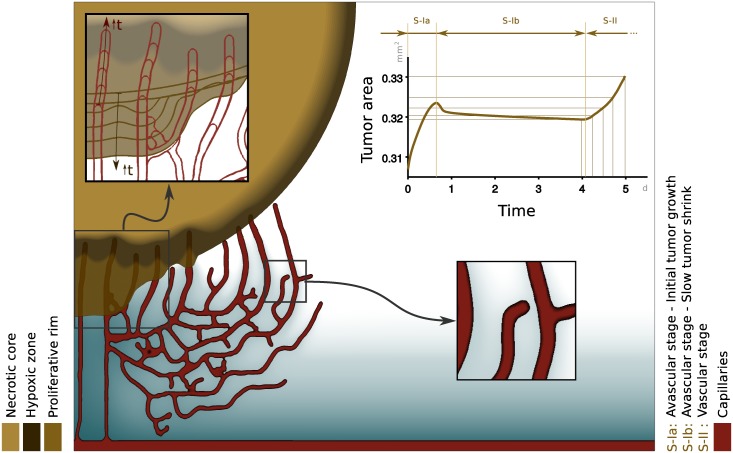
Simulation of vascular tumor. Hypoxic tumor cells (dark brown; see color scales in the bottom left corner) release tumor angiogenic factor (not shown in the plot for clarity) that promotes the growth of capillaries (red). The new vasculature brings additional nutrients (blue scale) to the tumor favoring its growth (see the tumor area evolution in the top right inset). The top left inset shows through contour lines the time evolution of the tumor and the capillaries that have penetrated it. The bottom right inset shows a tip endothelial cell that is about to anastomose with other capillary and the nutrient distribution in the surroundings of the neovasculature. As expected, the nutrient concentration decreases with the distance to the capillaries.

Now, we repeat the same computation using all the equations in our theory and allowing the tumor to trigger angiogenesis naturally as predicted by the model. Fig 6 shows the prediction of the model at time *t* = 5.1 days. The result clearly shows that the tumor quickly triggers angiogenesis, providing hypoxic cells with additional nutrients that can sustain their growth. To induce angiogenesis, hypoxic cells release TAF [see Eq (12)], which is not shown in the plot for clarity. The capillaries are represented in the figure by red areas, which delimit the zones in which *c* ≈ 1. The simulation clearly shows the experimental observation that angiogenesis occurs at much shorter time scales than tumor growth. In fact, the vascular network changes significantly in the same time lapse in which the tumor grows approximately 10% (see Fig 6). The top right inset shows the time evolution of the tumor area. This curve suggests three stages in tumor growth. At the beginning, the tumor grows slightly due to the nutrient that was initially in the tissue. Once this nutrient has been consumed, the tumor becomes hypoxic and starts to shrink, but also releases TAF which will later reach the capillaries and trigger angiogenesis. Once the newly-created capillaries are sufficiently close to the tumor, malignant cells start to receive nutrients and proliferate, giving rise to the third growth stage observed in the evolution of the tumor area. An important feature that the model predicts is that even after angiogenesis, a large part of the tumor is necrotic or hypoxic, as observed in experiments [17, 82]. We note, however, that there is also experimental evidence showing that the central necrosis disappears in small tumor spheroids [83]. In S1 Text and S1 Fig in the Supporting Information, we provide an additional numerical example that shows how our model reproduces this experimental observation, too.

In Fig 6, we have divided the tumor based on the amount of nutrient available. We distinguish three areas, namely, the necrotic core, the hypoxic zone, and the proliferative rim. These areas are represented using different brown hues (see the tumor color scale on the bottom left of Fig 6). The inset on the bottom right shows a TEC that stopped following the chemotatic cues because its filopodia detected a nearby capillary. As a consequence, the TEC altered its migration towards the capillary and will eventually anastomose with it. In addition, the inset shows how the nutrient is released by newly-created capillaries and diffuses throughout the surrounding tissue. The top left inset shows another important feature of vascular growth predicted by the model. It may be observed how the capillaries slowly penetrate the tumor while malignant cells proliferate in their surroundings giving rise to a phenomenon that resembles vessel cooption [84].

### Blockade of Dll4 Signaling Inhibits Tumor Growth

**Fig 7:**
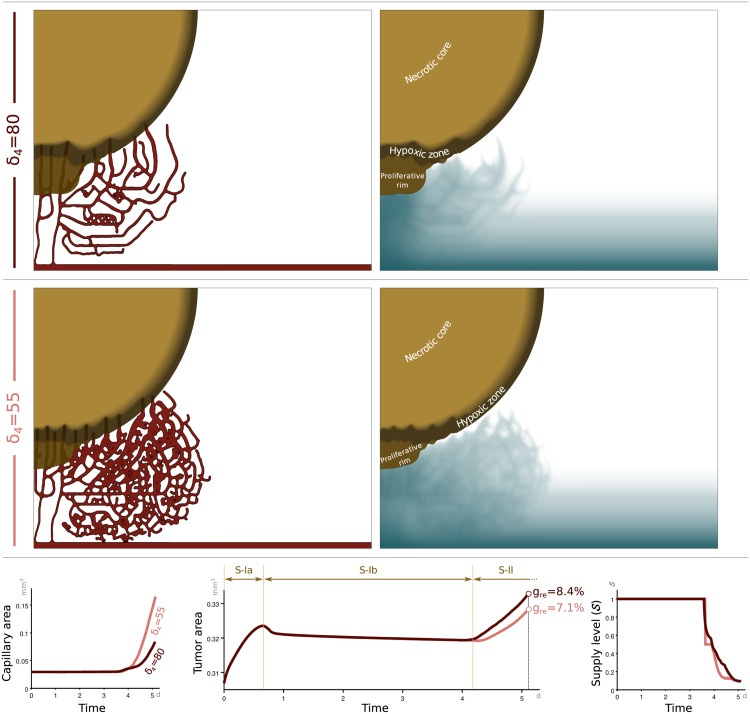
Simulation of tumor-growth reduction by blocking Dll4. The simulation in the top row shows snapshots of the tumor (brown scale), capillaries (red), and nutrient concentration (blue scale) for a typical value of the effective Dll4 distance, namely *δ*_4_ = 80. Note that on the right hand side panels we removed the capillaries to allow for a clearer observation of the nutrient distribution. The mid row shows the results of an analogous simulation in which the effective Dll4 distance has been reduced to *δ*_4_ = 55 to simulate the negative regulation of the Dll4 signaling pathway. As expected, the simulation shows a denser vascular network. The bottom left panel quantitatively illustrates this point. The denser vascular network, however, does not lead to faster tumor growth, but the opposite (see the bottom central panel). This phenomenon has been observed experimentally and is a consequence of the reduced transport functionality of the vascular network. In our model, the transport capacity is measured by the quantity S [see Eq (7)], whose time evolution is shown in the bottom right panel.

In this section, we observe the effects of down-regulating the Delta/Notch pathway on vascular tumor growth by using the proposed model. As stated in [18, 85–88], blockade of the Delta/Notch pathway resulted in remarkably increased tumor vascularity, associated with enhanced angiogenic branching. However, this increased vascularity was non-productive due to poor perfusion. To show that our model reproduces this observation, we perform two computations. The first one, shown in the top panels of Fig 7, uses again the parameters reported in Table 1. The second one, plotted in the mid panels of Fig 7, uses the same values for all the parameters except the Dll4 effective distance, which takes the value *δ*_4_ = 55. Thus, we model the down-regulation of Dll4 by allowing TECs to be created closer to each other. It may be easily observed that when *δ*_4_ is smaller, the vascular network is denser. This is also reflected in the bottom-left subplot, which represents the time evolution of the capillary area. As indicated before, we use the quantity S, defined in Eq (7), as a crude measure of the vascular network functionality. The bottom-right subplot shows that S=1 initially, and until new capillaries grow (*t* ∼ 3 days). Then, the value of S gradually decreases, reducing the effective nutrient supply in capillaries as shown in Eq (6). At a given time, the value of S is smaller when the Delta/Notch pathway is down regulated, as expected. As a measure of tumor growth, we use the quantity *g*_*re*_ = (*A*_*t*_ − *A*_0_)/*A*_0_, where *A*_*t*_ is the tumor area at time *t*, while *A*_0_ is the initial tumor area. In the mid panel of the bottom row of Fig 7, we plot the time evolution of the tumor area for both values of *δ*_4_. The curves have a similar structure to that shown in Fig 6, with three different regimes. First, the tumor undergoes a slight avascular growth until the nutrient given in the initial condition is consumed. Then, the tumor becomes hypoxic, releases pro-angiogenic factors, and shrinks slightly until the newly-created capillaries are able to provide nutrients to the tumor. After that, vascular growth begins. The plot also shows the final value of the relative growth *g*_*re*_, showing that the tumor grows more slowly when Dll4 is blocked, even if the vascular network is denser. This confirms that our model predicts the experimental findings in [18, 85].

## Discussion

We presented a model for coupled tumor growth and angiogenesis. The model resolves the capillaries to full scale, without introducing upscaled quantities such as, for example, microvascular density. Our computations show that the model can be used to study both avascular and vascular growth. In the avascular case, that is, when the tumor is not able to promote angiogenesis, our model predicts a slowly-shrinking, almost-dormant tumor due to its lack of access to nutrient. However, in the vascular case, the model naturally predicts the angiogenesis switch. Under this circumstances, the tumor has the ability to create a new exclusively-dedicated vascular network by means of induced angiogenesis that facilitates cancerous cell fast replication. Furthermore, the time evolution of the tumor area reveals the presence of an avascular stage the precedes the fully development of the new capillaries, during which the lesion presents a slow size reduction. It is only when the nutrient reaches the tumor through the new capillaries that the angiogenesis switch is effective and the vascular stage starts, triggering a fast lesion growth. Our theory also predicted a known phenomenon in vascular growth which consists of hindering tumor growth by negatively regulating the Delta/Notch signaling pathway. Our simulations show how a defective vascular network with an increased number of capillaries counter-intuitively decelerates rather than boosts tumor growth.

We believe our work opens new possibilities for those interested in exploring computationally complex scenarios in which tumors and capillaries interact. A significant problem that could be explored with our model is that of vessel cooption, which was observed experimentally [84]. In vessel cooption, the usual avascular to vascular transition enabled by angiogenesis is altered. Indeed, some tumors do not begin as avascular masses, but initially grow coopting existing blood vessels. The coopted host vasculature does not experience angiogenesis immediately, but regresses producing hypoxia and necrosis in the tumor. Eventually, the tumor triggers angiogenesis and continues its growth.

Finally, an important research topic that we plan to explore in the future is the application of our model to larger systems, ideally including three-dimensional, patient-specific tissue geometries and parameters.

## Supporting Information

S1 Text Disappearance of central necrosis in small tumor spheroids. Additional numerical example that reproduces the experiments reported in [83], where the authors observe the disappearance of the central necrosis after vascularization of tumor spheroids of size approximately 3 times smaller than those considered in the main text of the paper.
(PDF)

S1 Fig Disappearance of central necrosis in small tumor spheroids. Top-left: Geometry of the computational domain. Top-right: Time evolution of the necrotic area. Bottom-left: Initially, the necrotic core grows after angiogenesis. Bottom-right: Later on, the necrotic core shrinks until its complete disappearance. The two bottom sub-figures are both zoomed in from the dashed rectangle shown in the top-left panel. The tumor is plotted in solid brown color and the capillaries are shown in solid red color.
(EPS)
