# Supplementary material for: A Mathematical Model Coupling Tumor Growth and Angiogenesis
Source: PLoS One. 2016 Feb 18;11(2):e0149422. doi: 10.1371/journal.pone.0149422 (PMC4758654; doi:10.1371/journal.pone.0149422)
Supplement: S1 Text — Additional numerical example that reproduces the experiments reported in [83], where the authors observe the disappearance of the central necrosis after vascularization of tumor spheroids of size approximately 3 times smaller than those considered in the main text of the paper. (PDF) [file pone.0149422.s001.pdf]

Text S1

Disappearance of central necrosis in small tumor spheroids

It is currently believed that a large part of most tumors remains necrotic or hypoxic even after angiogenesis [1, 2] and our model successfully predicts this behavior. However, there is also experimental evidence showing that the central necrosis disappears after vascularization in small tumor spheroids [3]. Our hypothesis is that the neovasculature can penetrate deep enough into small tumors so as to restore normoxia, while the central part of larger tumors remains rather inaccessible to oxygen. Here, we try to reproduce the experiments reported in [3], where the authors observe the disappearance of the central necrosis after vascularization of tumor spheroids of size ~ 3 times smaller than those considered in the main text of the paper. Therefore, we perform a simulation on a rectangular tissue of $1350\,\mu\text{m}\times 775\,\mu\text{m}$ using the parameters reported in Table 1 in the main text of the paper. We place an initial circular tumor at the center of the domain and two horizontal capillaries in the bottom and top boundaries. The capillaries are supposed to be initially straight with a constant width of $25\,\mu\text{m}$. Boundary conditions are also taken to maintain symmetry, so that we can perform the computations on a quarter of the domain (see S1 Fig in the Supporting Information). The mesh is composed of 512×256 quadratic elements. The radius of the initial tumor is the same as in reference [3], namely, $\sim 200\,\mu\text{m}$. The initial source of nutrient is the capillary at the bottom, where σ takes the value 1. As in the previous simulations, we assume that, initially, the tumor has a circular necrotic core concentric to the tumor and with a radius $R_n = 0.35 R_t$, where $\sigma = 0$. Everywhere else in the tissue, $\sigma = 0.45$.

S1 Fig in the Supporting Information shows the results of this experiment. The top-left panel displays the initial configuration, while the top-right one shows the time evolution of the necrotic area. It is observed that the necrotic area increases initially (bottom-left panel), until the newly-created capillaries penetrate deep into the tumor and are able to provide nutrients that restore normoxia (bottom-right panel).

In all, this example shows how the model is able to reproduce another experimental observation.

References

1. Harris AL. Hypoxia—a key regulatory factor in tumour growth. Nat Rev Cancer. 2002;2(1):38–47.

2. Pennacchietti S, Michieli P, Galluzzo M, Mazzone M, Giordano S, Comoglio PM. Hypoxia promotes invasive growth by transcriptional activation of the met protooncogene. Cancer cell. 2003;3(4):347–361.

3. Wartenberg M, Dönmez F, Ling FC, Acker H, Hescheler J, Sauer H. Tumor-induced angiogenesis studied in confrontation cultures of multicellular tumor spheroids and embryoid bodies grown from pluripotent embryonic stem cells. The FASEB Journal. 2001;15(6):995–1005.
